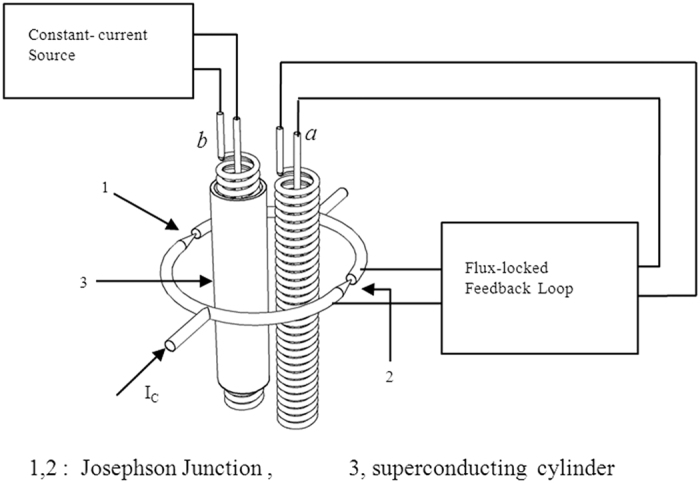# An experimental proposal to test the physical effect of the vector potential

**DOI:** 10.1038/srep19996

**Published:** 2016-01-29

**Authors:** Rui-Feng Wang

**Affiliations:** 1 Department of Physics, Beijing Jiaotong University, Beijing, 100044, China

## Abstract

There are two interpretations of the Aharonov–Bohm (A–B) effect. One interpretation asserts that the A–B effect demonstrates that the vector potential is a physical reality that can result in the phase shift of a moving charge in quantum mechanics. The other interpretation asserts that the phase shift of the moving charge results from the interaction energy between the electromagnetic field of the moving charge and external electromagnetic fields. This paper briefly reviews these two interpretations and analyzes their differences. In addition, a new experimental scheme is proposed to determine which interpretation is correct.

In classical electrodynamics, the electric field **E** and magnetic field **B** are physical fields; however, the scalar potential *φ* and vector potential **A** are the mathematical fields used to calculate **E** and **B**. As *φ* and **A** themselves do not have any observable effects under this interpretation, **E** and **B** are considered to be more fundamental entities than *φ* and **A**. However, in quantum mechanics, the scalar potential *φ* and vector potential **A** appear in the Schrödinger equation instead of the electric field **E** and magnetic field **B**. Therefore, some physicists asserted that *φ* and **A** are more fundamental than **E** and **B** in quantum mechanics^1^. Aharonov and Bohm predicted a new effect wherein the phase of a moving charge could be changed in regions of non-zero *φ* and **A** even if **E** and **B** are both zero^2^. This effect, which is known as the Aharonov–Bohm (A–B) effect, includes electric and magnetic A–B effects (in fact, the magnetic A–B effect was first predicted by Ehrenberg and Siday^3^ in 1949). The magnetic A–B effect has been extensively studied both experimentally^4^,^5^,^6^,^7^,^8^,^9^,^10^,^11^ and theoretically^12^,^13^,^14^,^15^,^16^,^17^,^18^,^19^. The existence of this effect has been supported by several experiments^4^,^5^,^6^,^7^,^8^,^9^,^10^,^11^, especially one performed by Tonomura in 1986^11^. However, the electric A–B effect has been studied much less^20^,^21^,^22^,^23^,^24^,^25^.

Interpretations of the A–B effect can be roughly classified into two types. The first interpretation asserts that the effect is caused by the electromagnetic potentials of the excluded electromagnetic fields, i.e., the electromagnetic potentials **A** and *φ* can result in some observable phenomena in quantum mechanics even though they are only mathematical fields in classical physics. This interpretation is referred to as “the interpretation of electromagnetic potentials” in this paper^1^,^2^,^12^. The second interpretation asserts that the effect is caused by the energy of interaction between a moving electron’s electromagnetic fields and the excluded electromagnetic fields. This interpretation is referred to as “the interpretation of interaction energy” in this paper^15^,^18^,^19^,^25^.

Since the differences between these two interpretations regarding the electric A–B effect have been discussed in literature^25^, this paper only focuses on the magnetic A–B effect. Disparities between the two interpretations regarding the magnetic A–B effect will be briefly introduced without prejudice in the second part of this paper. In the third part, we will provide some comments on the arguments. In the fourth part, a new experimental proposal to judge which interpretation is correct will be presented. The fifth part presents the conclusions.

## Differences Between the Two Interpretations

Suppose there is a long, straight solenoid in space. A coherent beam of electrons is split into two parts, each going on opposite sides of the solenoid but avoiding it. Then, these two beams are brought together to interfere with each other. Aharonov and Bohm^2^ predicted that the interference pattern between the split electron beams is determined by the magnetic flux through the solenoid. The phase difference Δ*ϕ* between the beams is given as


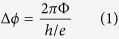


where Φ is the magnetic flux through the solenoid. This prediction has been supported in several experiments^4^,^5^,^6^,^7^,^8^,^9^,^10^,^11^. Although the existence of the magnetic A–B effect is not currently in question, there is as of yet no general agreement on what actually causes the phase difference Δ*ϕ* between the two electron beams. Different interpretations yield different answers.

### The Interpretation of Electromagnetic Potentials

This interpretation insists that the factor causing the phase difference Δ*ϕ* can only be the vector potential **A** because only **A** is non-zero in the region outside of the solenoid; all other quantities are zero in this region and, hence, cannot affect the phases of the electron waves.

### The Interpretation of Interaction Energy

This interpretation insists that the factor causing the phase difference Δ*ϕ* is not the vector potential **A** but rather the interaction energy *E*′ between the moving electron’s magnetic field **B**_2_ and the magnetic field **B**_1_ produced by the solenoid. Here, the interaction energy *E*′ between these two fields is determined by^18^


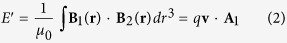


where **A**_1_ is the vector potential describing the magnetic field **B**_1_ produced by the solenoid, *q* and **v** are the charge and velocity of the moving charge, respectively.

The interpretation of interaction energy asserts that the interaction energy *E*′ between fields **B**_1_ and **B**_2_ causes the magnetic A–B effect. Although *E*′ can be described in terms of the vector potential **A**, the vector potential **A** does not directly cause the magnetic A–B effect by itself.

### Differences between the two interpretations

**Figure 1:**
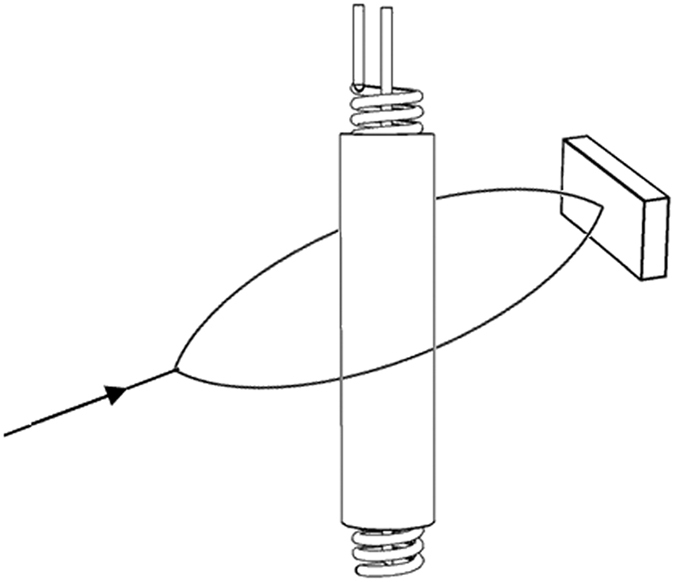


In most experiments^4^,^5^,^6^,^7^,^8^,^9^,^10^, both vector potential **A** and the interaction energy *E*′ simultaneously exist; furthermore, the same conclusions are reached regardless of which is assumed, making it impossible to determine which interpretation is correct in these experiments. However, the two interpretations will result in different conclusions in one situation: it is that the magnetic flux is coated by a superconducting cylinder and the charges move outside the cylinder, as depicted in Fig. 1. Because of the Meissner effect^26^, the superconducting cylinder can completely shield the magnetic field produced by the moving charge outside it; therefore, the magnetic field of the moving charge cannot overlap with the magnetic field produced by the solenoid. Thus, the interaction energy *E*′ between these two magnetic fields must be zero in this situation; however, the vector potential **A** still exists outside the superconducting cylinder. If the interpretation of electromagnetic potential is correct, the A–B effect should be observed in this situation; however, if the interpretation of interaction energy is correct, the A–B effect should not be observed. Using this experimental method, we can attempt to determine which interpretation is correct. This experimental method was first proposed in ref. 13.

Using this method, Tonomura performed a similar experiment in 1986^11^ on tiny toroidal magnets covered with superconductors. In the experiment^11^,^27^, the phase difference Δ*ϕ* between an electron wave passing through the hole of a toroid sample and a wave passing outside the toroid was measured using an interferogram. It was found that the electron interference pattern changed with the magnetic flux enclosed by the superconducting layer, indicating that the A–B effect could be observed even though the magnetic flux was enclosed by the superconducting cylinder. Based on this, most physicists began to believe the interpretation of electromagnetic potentials, causing a loss of interest in the interpretation of interaction energy.

However, it has recently been argued^18^,^19^ that the superconducting film in the experiment by Tonomura could only confine the magnetic flux within it but could not shield the magnetic field produced by the high speed electron beams used in the experiment. Therefore, the interaction energy between two magnetic fields still exists in this experiment. Hence, it was asserted that this experiment cannot show that the interpretation of interaction energy is wrong. A detailed discussion of the problem can be found in ref. 19 wherein it is further asserted that only the interpretation of interaction energy is consistent with the uncertainty principle.

## Comments on the Dispute

The key problem with respect to the A–B effect is determining which factor affects the phase of moving charges. If the phase change of a moving charge is caused by the interaction energy of electromagnetic fields, then the A–B effect is an ordinary interference phenomenon and the vector potential **A** and scalar potential *φ* are still mathematical fields in quantum mechanics that cannot cause any observable effect by themselves. However, if the phase change of the moving charge is caused by the vector potential **A** and the scalar potential *φ* but not by the interaction energy of the electromagnetic fields, then, the A–B effect shows that the vector potential **A** and scalar potential *φ* are physical realities in quantum mechanics instead of the mathematical fields in classical electrodynamics, for they can result in some observable effect by themselves. This change would imply a great difference in the basic concept of gauge fields between quantum mechanics and classical physics. Therefore, clarifying which factor causes the A–B effect is very important.

To date, only the experiment conducted by Tonomura in 1986 has addressed the issue. If the superconducting film could shield the magnetic field of the moving electrons in this experiment, then, to our knowledge, this experiment is the only experiment that demonstrates the magnetic A–B effect due to the vector potential **A** and not due to the interaction energy *E*′. If the superconducting film could not shield the magnetic field of the moving electrons, then no experiment conducted to date has been able to distinguish whether the magnetic A–B effect is caused by the vector potential **A** or by the interaction energy, for no other experiment was performed to clarify this problem after the experiment by Tonomura. Whether the vector potential **A** and scalar potential *φ* are physical realities or mathematical fields in quantum mechanics is a very fundamental problem that requires additional experimental investigation. Even though there was no dispute about the experiment by Tonomura, only one experiment is not sufficient for such an important conclusion. It is necessary to design new experiments to study the problem and verify the conclusion.

## A New Experimental Proposal

To avoid the disputes outlined above concerning the experiment conducted by Tonomura, we propose a new experimental scheme. Our new experiment employs a dc-superconducting quantum interference device (SQUID)^6^.

A dc-SQUID is a superconducting loop with two Josephson junctions. The critical current *I*_*C*_ passing through the SQUID is determined by the phase differences of Cooper pairs across the Josephson junctions, which are related to the flux Φ through the superconducting loop. Though the magnetic field is zero in the region where the superconducting loop is located, the critical current *I*_*C*_ passing through the SQUID is determined by the following equation:


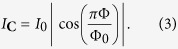


where 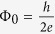.

Obviously, the A–B effect, which is the effect exerted by a magnetic field on the phase of moving electrons, can be explored in the context of a SQUID. Thus, the SQUID can duplicate the A–B effect with the only difference being that the moving electrons in the A–B effect are single electrons, whereas the moving electrons in the SQUID are Cooper pairs.

In terms of SQUID dynamics, an important question is “does the critical current *I*_*C*_ depend on the vector potential **A** describing the magnetic flux Φ through the SQUID or does it depend on the interaction energy between the magnetic flux Φ through the SQUID and the magnetic field produced by the SQUID? ” This question can be answered with the following experiment.

The experimental arrangement is depicted in Fig. 2. The dc-SQUID used in this experiment is a “point contact” device, which can be fabricated according to the description in Fig. 7(b) in ref. 28 with Nb as the fabricating material. The diameter of the hole enclosed by the superconducting loop is about 6–7 *mm*. Two similar solenoids, denoted as *a* and *b*, are enclosed by the superconducting loop of the dc-SQUID. For simplicity, we assume that solenoids *a* and *b* have identical dimensions: length *l* = 50 *mm* and diameter *d* = 1 *mm*. If each solenoid contains 500 coils, i.e., 10 turn per *mm*, then the flux Φ through one turn of a solenoid equals Φ_0_ when a current of 0.21 μA passes through the solenoid wire. Solenoid *a* is connected to a flux-locked feedback loop and used as a negative feedback coil^29^. The function of the flux-locked feedback loop is to keep the current *I*_*C*_ of the SQUID constant by changing the current *I*_*a*_ of solenoid *a*. Solenoid *b* is surrounded by an additional superconducting cylinder and connected to an independent constant-current source. In this experiment, the superconducting cylinder can be made of Sn, its inner diameter can be 1.5 *mm* and the outer diameter can be 3 *mm*. The Sn cylinder can completely shield the magnetic field produced by the dc-SQUID, for the working frequency of the dc-SQUID is very low (typically 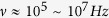, 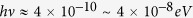), which is much less than the *kT*_*C*_ of Sn (The critical temperature *T*_*C*_ of Sn is about 3.72 *K*, and its *kT*_*C*_ is about 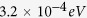)^19^,^30^.

Using a SQUID and cylinder fabricated as above, the experimental steps can be performed as follows:

Step 1. The dc-SQUID system and Sn cylinder are cooled to a temperature of 4.2 K, at which point the SQUID is in a superconducting state, but the Sn cylinder is in a normal state of conductivity. During this step, the flux-locked feedback loop is turned off and the current *I*_*a*_ in solenoid *a* remains zero. The current *I*_*b*_ in solenoid *b* is increased to a value ensuring that the flux through each turn of *b* is 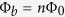 (for example, for 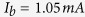, *n* = 5000). Although, according to eq. (3), the critical current *I*_*C*_ of the SQUID will change with Φ_*b*_, this change can be disregarded in this step.

Step 2. The flux-locked feedback loop is turned on, and then, the temperature of the system (including the SQUID, two solenoids, and the Sn cylinder) is decreased to 2.5 K. In this process, the critical current *I*_*C*_ of the SQUID will increase slightly with the temperature decreasing, but this will not affect the functioning of the flux-locked feedback loop. Because a temperature gradient will inevitably exist in the experimental system during temperature decreases, the Sn cylinder will be cooled into the superconducting state from one end to the other in a stepwise manner. At the same time, the magnetic field produced by the SQUID will be excluded from the Sn cylinder step by step because of the Meissner effect. In this process, the current *I*_*b*_ in the solenoid *b* remains unchanged, i.e., 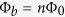 does not change, and therefore, the vector potential **A**_*b*_ describing the magnetic field through the solenoid *b* remains unchanged as well. However, the interaction energy *E*′ between the magnetic field produced by the SQUID and the magnetic field through solenoid *b* will become zero in a stepwise manner.

If the critical current *I*_*C*_ is dependent on the interaction energy *E*′ between the magnetic field produced by the SQUID and the magnetic field through its superconducting loop, then as the Sn cylinder is cooled into the superconducting state during the step 2, the current *I*_*a*_ in solenoid *a* will increase to compensate for the change of the interaction energy *E*′. After the entire Sn cylinder is cooled to the superconducting state, the magnetic flux Φ_*a*_ through solenoid *a* should equal Φ_*b*_ in solenoid *b*, i.e., 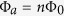. Only in this manner can the interaction energy *E*′ remain unchanged. After the Sn cylinder is cooled to the superconducting state, the total magnetic flux through the SQUID will be 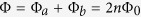.

If, on the other hand, the critical current *I*_*C*_ of the SQUID is dependent on the vector potential **A** describing the magnetic flux Φ through the SQUID, then *I*_*C*_ should remain constant throughout the process as Φ_*b*_ and **A**_*b*_ will remain unchanged. Therefore, it will be unnecessary to change the current *I*_*a*_ in solenoid *a* to keep *I*_*C*_ constant, and the magnetic flux Φ_*a*_ through each turn of solenoid *a* will remain zero, i.e., Φ_*a*_ ≡ 0. In this situation, after the entire Sn cylinder is cooled into the superconducting state, the total magnetic flux through the SQUID will be 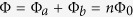.

Therefore, the above experimental result can clearly determine whether the key factor affecting the phase of the moving electrons is the electromagnetic potential **A** or the interaction energy *E*′ between the electromagnetic fields. Answering this problem can help us better understand the Schrödinger equation in electromagnetic fields.

## Conclusions

Determining the true cause of the A–B effect is related to how we understand the role of the electromagnetic potentials **A** and *φ* in the Schrödinger equation. Determining whether these electromagnetic potentials can cause observable phenomena by themselves in quantum mechanics is an important problem. Regardless of the answer, both interpretations need to be supported by numerous experiments. In this paper, we propose a new experimental method to study this question. Future experimental results based on our proposed methodology will help us better understand the basic properties of gauge fields.

## Additional Information

**How to cite this article**: Wang, R.-F. An experimental proposal to test the physical effect of the vector potential. *Sci. Rep.* **6**, 19996; doi: 10.1038/srep19996 (2016).

## Figures and Tables

**Figure 2:**